# Diagnostic automated algorithms in neurodevelopmental disorders: Focus on automatic motor assessment

**DOI:** 10.1192/j.eurpsy.2021.72

**Published:** 2021-08-13

**Authors:** T. Gargot

**Affiliations:** Child And Adolescent Psychiatry, CHRU de Tours, Tours, France

**Keywords:** motor assessment, neurodevelopment disorders, Digital phenotyping, autism

## Abstract

Difficulties in motor development are frequent and impairing. However, the assessment of these motor learning skills is difficult and limits early stage rehabilitation. Electronic sensors and algorithms can help to measure motor difficulties more easily and objectively. We will present a systematic review detailing these methods and challenges in Autism Spectrum Disorders (ASD). Electronic tablets, give access to handwriting features that are not usually evaluated in classical assessments. We describe how such digital features (in static, dynamic, pressure, and tilt domains) allow diagnosing dysgraphia and how they evolve during children development. From a finer analysis, three different clusters of dysgraphia emerge, longitudinal studies will allow to underline different patterns of development that seemingly require tailored remediation strategies. However, those digital features are not used in the context of conventional pen and paper therapies. It is possible to engage children with typical development in handwriting exercises by asking them to teach a robot to write. We implemented a long-term case study (20 sessions, 500 minutes in total) observing a child with severe Developmental Co-ordination Disorder who did not progress anymore with a classic pen and paper approach by enriching this setup with various training activities using real-time feedback loops (on tilt, pressure, dynamic, pauses). We show how this new method tackles previous child’s behaviour avoidances, boosting his motivation, and improving his motor and writing skills. This talk demonstrates how new motor digital features allow the implementation of innovative motor remediation interventions, which rely on fostering children’s personal characteristics and adaptation skills.

**Disclosure:**

The work presented in this talk was conducted during a PhD in computer science (funded by APHP and Paris 8 University, France). A company was funded by a PhD collaborator, dynamico.ch. The presenter does not have consultancy or financial connection with d

